# Resistive-Based Gas Sensors Using Quantum Dots: A Review

**DOI:** 10.3390/s22124369

**Published:** 2022-06-09

**Authors:** Ali Mirzaei, Zoheir Kordrostami, Mehrdad Shahbaz, Jin-Young Kim, Hyoun Woo Kim, Sang Sub Kim

**Affiliations:** 1 Department of Materials Science and Engineering, Shiraz University of Technology, Shiraz 71557-13876, Iran; mirzaei@sutech.ac.ir; 2 Department of Electrical and Electronic Engineering, Shiraz University of Technology, Shiraz 71557-13876, Iran; kordrostami@sutech.ac.ir; 3 Department of Materials Science and Engineering, Urmia University, Urmia 5766-151818, Iran; m.shahbaz@urmia.ac.ir; 4 Department of Materials Science and Engineering, Inha University, Incheon 22212, Korea; piadote@naver.com; 5 Division of Materials Science and Engineering, Hanyang University, Seoul 04763, Korea; 6 The Research Institute of Industrial Science, Hanyang University, Seoul 04763, Korea

**Keywords:** Quantum dots (QDs), gas sensor, toxic gas, sensing mechanism

## Abstract

Quantum dots (QDs) are used progressively in sensing areas because of their special electrical properties due to their extremely small size. This paper discusses the gas sensing features of QD-based resistive sensors. Different types of pristine, doped, composite, and noble metal decorated QDs are discussed. In particular, the review focus primarily on the sensing mechanisms suggested for these gas sensors. QDs show a high sensing performance at generally low temperatures owing to their extremely small sizes, making them promising materials for the realization of reliable and high-output gas-sensing devices.

## 1. Resistive-Based Gas Sensors: Basics

Air pollution is a global problem that caused ~4.9 million premature deaths in 2017 [1]. The human olfactory system is highly sensitive and can discriminate different odors. On the other hand, some dangerous gases are odorless. In some cases, the extremely low concentration of gases is not detectable by the human olfactory system. Furthermore, in many places, humans are not present or cannot be present to detect the odor of gases. Thus, sensitive devices of small size and high performance are needed to detect various toxic gases and vapors reliably [2]. Some techniques, such as ion chromatography and gas chromatography, require multi-step laboratory procedures. In addition, they are expensive, bulky, and cannot offer online signals [3,4]. 

There are various types of gas sensors, including surface acoustic waves [5], mass-sensitive [6], infrared [7], and optical [8], based on different materials and principles [9]. They are used for public security, environmental control, chemical quality control, safety in homes, automotive applications, air conditioning, and breath analysis for medical diagnoses [10,11]. Among the different gas sensors, conductometric sensing devices are popular owing to unique features, including (i) low cost, (ii) ease of fabrication and use, (iii) high response, (iv) high stability, (v) easy integration into sensor arrays, and (vi) simple operation [12]. Bradeen and Bradeen were the first to discover the gas-sensitive influences on semiconducting germanium [13]. Seiyama et al. [14] reported the first metal oxide gas sensor based on ZnO for toluene, CO_2_, and propane sensing. Taguchi later patented a SnO_2_ gas sensor and soon commercialized it [15]. 

**Figure 1:**
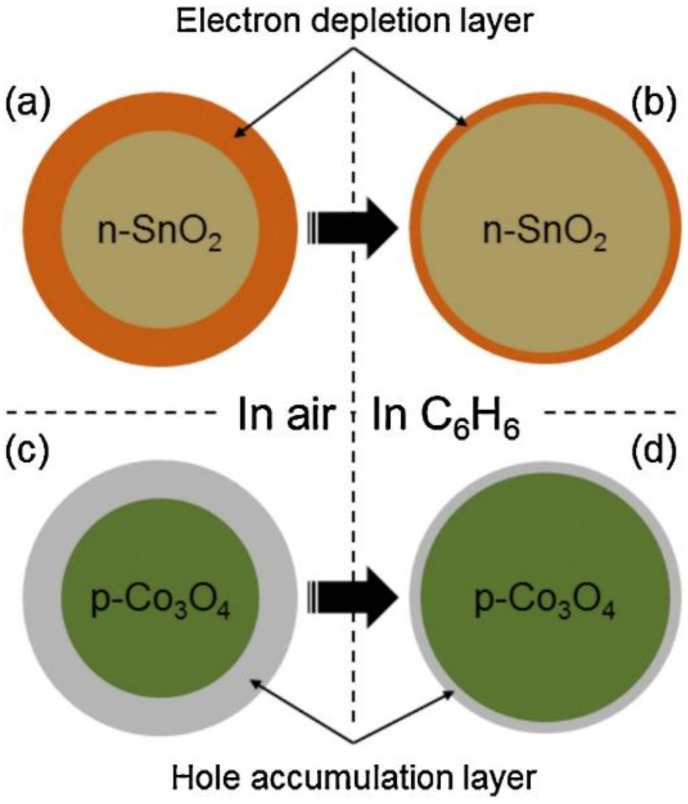
Sensing mechanism of n-SnO_2_ and p-Co_3_O_4_ (**a**,**b**) in air; (**c**,**d**) in C_6_H_6_, as an example of a reducing gas [17].

The principle of the sensing mechanism is modulation of the sensor resistance in different atmospheres [16]. Depending on the n-type or p-type nature of the sensing layer and the nature of the gas, the electrical resistance of sensing device changes in proportion to the amount of gas. In n-type materials, such as SnO_2_, an electron depletion layer initially exists in the air by adsorbed oxygen ions and subsequent exposure to a reducing gas. The liberated electrons return to the surface of the sensing layer, narrowing the width of the electron depletion layer. Therefore, they contribute to the sensor signal. For p-type materials, a hole accumulation layer exists initially in the air. The width of this layer decreases in a reducing gas medium, leading to an increase in sensor resistance. Figure 1 shows the mechanisms for n- and p-type gas sensors when a reducing gas is present [17]. 

Therefore, by tracking the resistance variations, a calibration curve can be drawn and used for applications [18]. Some shortages of resistive-based gas sensors are low selectivity and high sensing temperature [19]. The performance of these types of gas sensors can be improved using a range of methods, such as the formation of p-n heterojunctions [20], noble metal decoration [21], doping [22], UV irradiation [23], morphology engineering [24,25], and decrease of particle size. 

**Figure 2:**
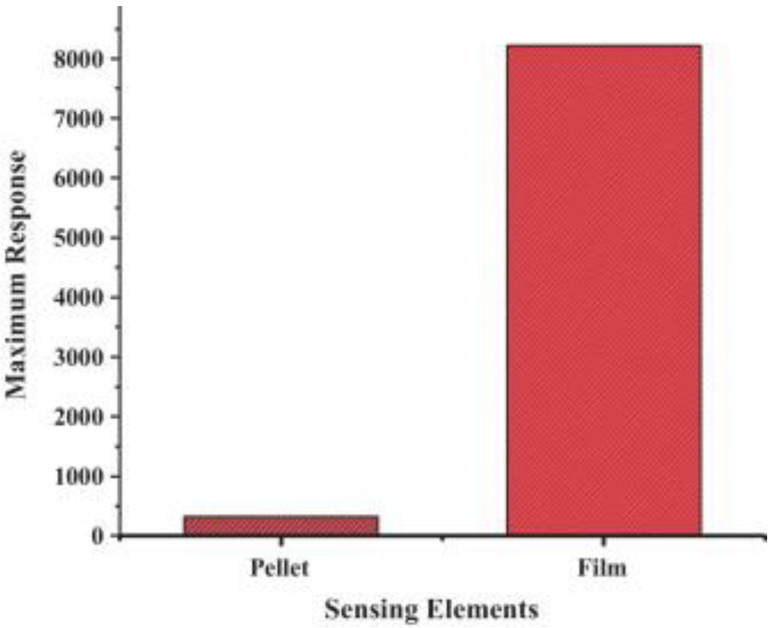
Response of NdFeO_3_ pellet and film gas sensors to LPG [27].

**Figure 3:**
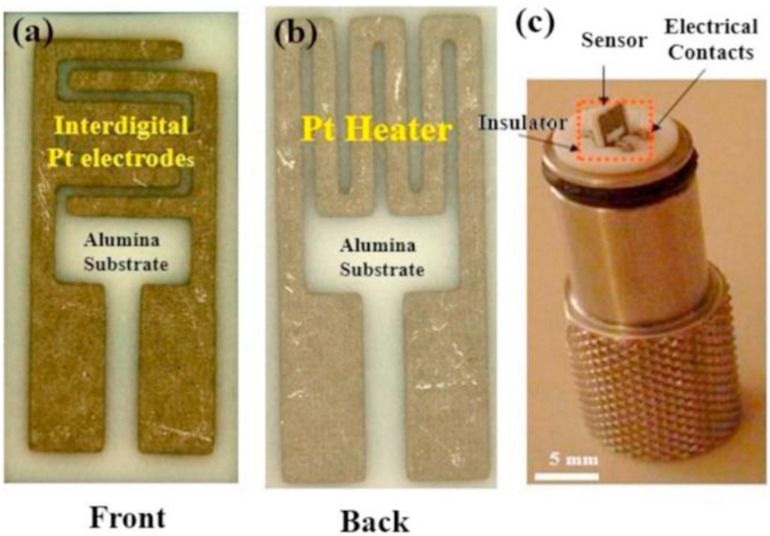
(**a**) Front; (**b**) back sides of an alumina substrate for gas sensing studies; (**c**) Sensor holder [29].

Generally, resistive-based gas sensors are fabricated by depositing a thin or thick film over an interdigitated insulator substrate [26]. The pellet form is not efficient as much of the bulk volume is inaccessible to the target gas, resulting in a lower response relative to either thin or thick film counterparts, as shown in Figure 2 [27]. Electrodes are used to provide an electrical signal for the electrical device. Sometimes a heater is incorporated in the backside of the substrate to offer the necessary temperature for operation [28]. Figure 3 presents the front and back sides of an alumina substrate equipped with electrodes and heaters in the front and back sides, respectively [29]. 

## 2. Quantum Dots: Definition and Applications

**Figure 4:**
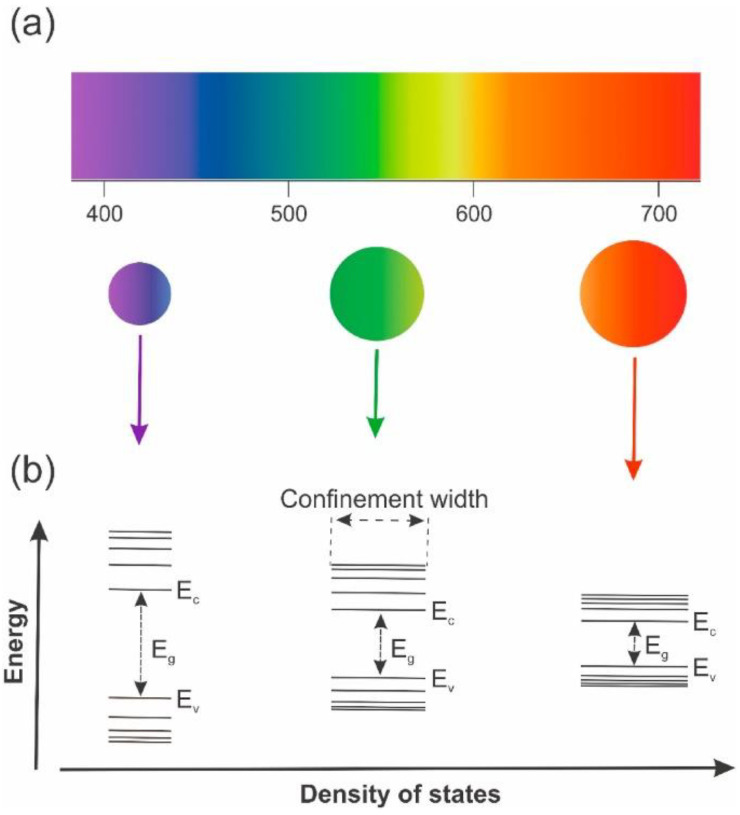
Schematic of (**a**) emission spectra QDs with different sizes; (**b**) the QDs size effect on their bandgaps [33].

Quantum dots (QDs) are unique semiconductor materials with exceptional tunable band gaps, functionalizable properties, and high surface areas [30]. By definition, QDs are nanocrystals, and their excitons and the motion of charge carriers are confined in all dimensions because of their ultrafine sizes [31,32]. Therefore, depending on the size of the particles, the energy difference between energy bands is changed in QDs. The bandgap of QDs can be tuned by modifying their sizes [33], as shown in Figure 4.

The exiton Bohr radius (r_B_) of some QDs are presented in Table 1. It can be calculated as follows [34]:

(1) $$r_{B}=\frac{\varepsilon h^{2}}{\mu_{eff}\pi e^{2}}$$

where $\varepsilon =\varepsilon_{r}\varepsilon_{0}$, *h* is Plank constant (6.62 × 10^−34^ m^2^ kg/s), and $\mu_{eff}$ is reduced effective mass of electron-hole pair given by Equation (2):

(2) $$\mu_{eff}=\frac{m_{h}^{*}m_{e}^{*}}{m_{h}^{*}+m_{e}^{*}}$$

where $m_{h}^{*}\;and\;m_{e}^{*}$ are electron and hole effective masses, respectively.

QDs are used for infrared photodetectors [42], solar cells [43,44], light-emitting diodes [45], as well as gas sensors [46]. For a detailed explanation about the synthesis techniques for different types of QDs, the readers can refer to [33].

## 3. Resistive-Based Gas Sensors Based on QDs

### 3.1. Pristine Metal Oxide and Metal Sulfide Quantum Dot Gas Sensors

#### 3.1.1. SnO_2_-Based Gas Sensors

SnO_2_ is a widely used material for sensing studies [47] because of its low price, good stability, and high mobility of electrons [48]. In this direction, Xu et al. [49] investigated the grain size effects in SnO_2_ gas sensors and reported that the gas-sensing features of SnO_2_ were enhanced by reducing the grain sizes. In particular, the sensing properties were increased when the grain size was comparable to the Debye length. Liu et al. [50], prepared SnO_2_ QDs (2.0–12.6 nm) and reported that the sensing response was significantly increased when the grain size was close to the Debye length of SnO_2_. 

**Figure 5:**
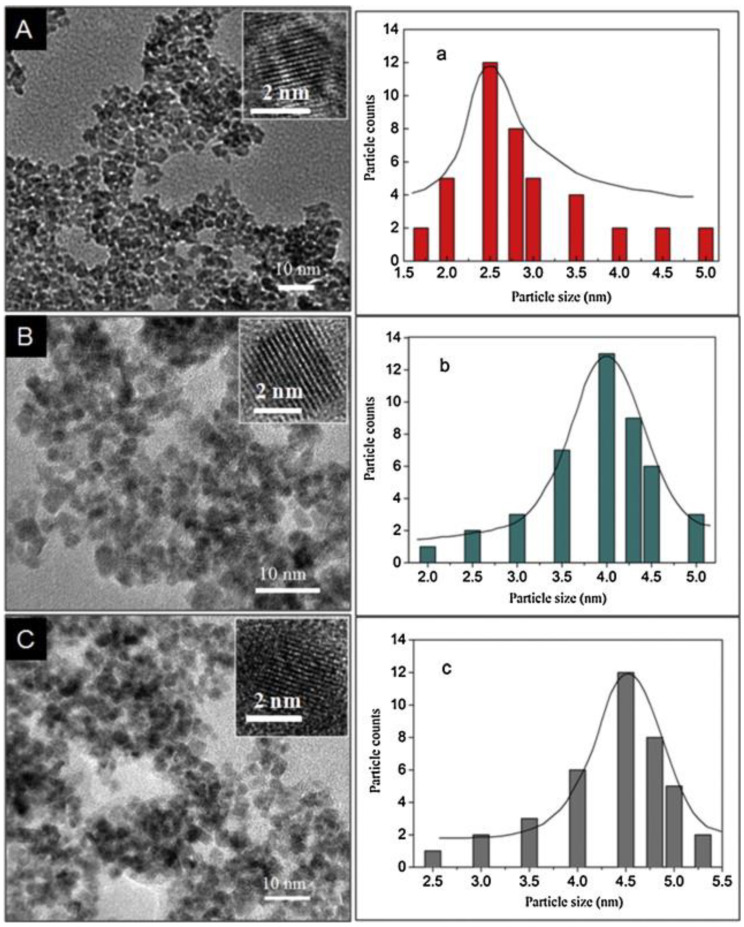
(**A**–**C**) TEM and HRTEM (insets) micrographs, and (**a**–**c**) relevant size distributions of SnO_2_ QDs prepared by hydrothermal synthesis [52].

The quantum size effects appear when the size of the SnO_2_ nanoparticles (NPs) is about 1–10 nm [51]. Du et al. [52] prepared SnO_2_ QDs via a hydrothermal route. By varying the amounts of alkaline reagent, the size of SnO_2_ QDs was adjusted to 2.5 ± 0.3 nm, 4.0 ± 0.3 nm, and 4.5 ± 0.3 nm (Figure 5). 

A previous study reported that at 240 °C, the response of SnO_2_ QDs to trimethylamine (TEA) increased with a decreasing SnO_2_ QDs size. First, because the size range of gas sensors was close to the Debye length of SnO_2_ and smaller than twice the thickness of the electron depletion layer (EDL), the entire crystal became depleted from electrons. Hence, subsequent exposure to TEA and the huge amount of resistance modulation causes a strong response on the gas sensors. Second, with further increases in size, the quantum confinement effect becomes more evident, and the surface defects increase. Thus, the highest responses to TEA were observed in a sensor with the smallest grain sizes. 

**Figure 6:**
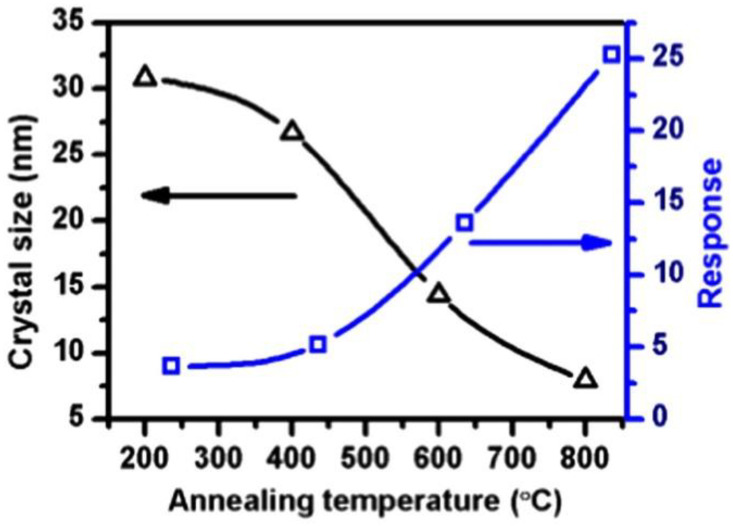
Sensing response and crystalline size of the SnO_2_ samples as a function of the annealing temperature [53]. Black line with triangle symbols shows variations of crystal size with annealing temperature and blue line with square symbols shows variations of response with annealing temperature.

Generally, high temperatures, complex organic solutions, and long reaction times are needed to prepare SnO_2_ QDs with ultra-small sizes. On the other hand, He et al. [53] reported a facile, room temperature precipitation method to synthesize ~2.5 nm SnO_2_ QDs. SnO_2_ QDs with different sizes were synthesized without needing a capping agent or an organic solvent or annealing at different temperatures. As shown in Figure 6, SnO_2_ QDs showed an enhanced response to ethanol gas relative to SnO_2_ NPs. The SnO_2_ QDs with a small size of 3.7 nm revealed a strong response to 30–50 ppm ethanol at 200 °C with fast response (1 s) and recovery (1 s) times. The strong response was related to the complete depletion of SnO_2_ QDs from electrons in air and subsequent resistance variation in the presence of ethanol. 

**Figure 7:**
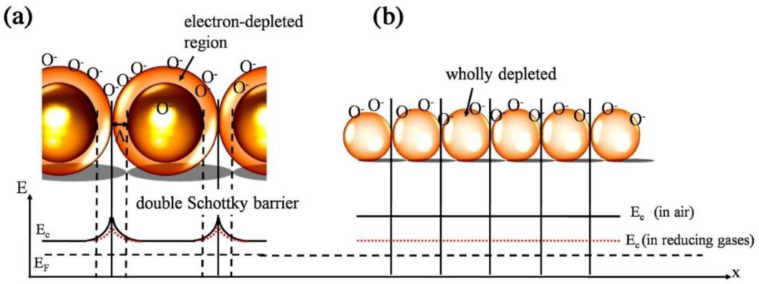
Sensing mechanism of SnO_2_ QDs. (**a**) formation of double Schottky barriers; (**b**) energy levels in SnO_2_ QDs when the grains are smaller than the thickness of the space charge layer [54].

Zhu et al. [54] synthesized SnO_2_ QDs (5–10 nm) by a microwave (MW)-assisted wet chemical method at 160 °C and subsequent annealing at 400 °C. In polycrystalline SnO_2_ grains, double Schottky barriers form between two neighboring grains in air and the motion of electrons is restricted in air (Figure 7a). Thus, the resistance is high in the air. In reducing gas atmosphere, the height of barriers decreases, increasing the conductance. When the particle size is smaller than the EDL thickness, the electron-depleted regions overlap (Figure 7b). In the case of SnO_2_ QDs, the whole SnO_2_ crystals become electron-depleted in air, and a ‘flat-band’ condition was expected. The energy difference between the conduction band (E_c_) and Femi level (E_F_) is increased. In a reducing gas atmosphere, the electrons return to the SnO_2_ QD surface, and the whole grains become more conducting than in the air, and an enhancement of gas sensitivity is expected. 

Colloidal QDs (CQDs) are semiconductor nanocrystals dispersed in solution. Solution processability can be obtained using long-chain ligands, such as oleic acid (OA) or oleylamine (OLA) capping on the CQD surfaces [55]. Liu et al. [56] synthesized OA and OLA capped SnO_2_ CQDs for H_2_S sensing studies. As reported elsewhere [57], these capping agents have long carbon chains that generate insulating barriers between CQDs and hinder efficient gas adsorption and carrier transport, resulting in poor gas sensing performance. Therefore, after spin coating the substrate, a surface ligand treatment was applied using AgNO_3_, NaNO_3_, NaNO_2_, KNO_2_, and NH_4_Cl to exchange long-chain surface-capping ligands. The ligand-treated samples showed a sensitive response to H_2_S gas. In particular, AgNO_3_-treated SnO_2_ CQD film revealed the strongest response to this gas. Characterization techniques approved the presence of Ag_2_O, which is a promising material for H_2_S gas sensing. At 70 °C, the AgNO_3_-treated SnO_2_ CQDs gas sensor indicated a high response to 29–50 ppm of H_2_S gas. In SnO_2_ CQD sensors, all the SnO_2_ CQDs become completely depleted from electrons because of their small sizes (~up to 10 nm). Hence, there are no surface barriers because there are no electrons in the entire crystal. Upon exposure to H_2_S gas, the Fermi level (E_Fg_) becomes much closer to the conduction band, resulting in a more conductive state. Therefore, the sensor response is related to the Fermi level shift, which depends on the amount of gas.

#### 3.1.2. ZnO QDs Gas Sensors

Semiconducting n-ZnO (E_g_ = 3.37 eV), which has high electron mobility and highly stable chemical and thermal properties, is popular for sensing studies [22,58]. Zhang et al. [59] prepared OA- capped ZnO CQDs using a facile colloidal method. OA capping was performed to avoid agglomeration. The OA-capped sensor revealed almost no response to H_2_S gas. The OA with long chains carbon limits electron flow and prevents gas molecules react with the ZnO surface. However, after treatment of the capping agent with different agents, the ZnCl_2_-treated gas sensor exhibited a response of 113.5 to 50 ppm of H_2_S gas. Nevertheless, its recovery was still poor. Upon annealing at 300 °C, the sensor showed a response of 113.5 with relatively fast recovery time. Forleo et al. [60] prepared ZnO QDs (2.5–4.5 nm) using a wet chemical method for gas sensing studies. At low temperatures, the sensor exhibited a high response to NO_2_ gas, whereas at T > 350 °C, strong responses to acetone and methanol were recorded. However, the recovery time was very long. 

#### 3.1.3. TiO_2_ QDs Gas Sensors

N-type semiconducting TiO_2_ is non-toxic, inexpensive, highly stable, and has unique electro–optical properties [61,62]. Liu et al. [63] prepared TiO_2_ QDs with a high surface area (315.74 m^2^/g). At 25 °C, it showed a good response of 7.8 to 10 ppm NH_3_ gas. The sensing mechanism was described based on the generation of EDL on TiO_2_ QDs.

#### 3.1.4. PbS QD Gas Sensors

Lead sulfide (PbS) is used widely for sensing studies [64,65,66]. Liu et al. [67] prepared PbS CQD sensors for NO_2_ gas-sensing applications. They compared the sensing output of the gas sensor on three substrates: Al_2_O_3_, PET, and paper at room temperature. The paper-based gas sensor revealed a high response of 21.7 to 50 ppm of NO_2_, whereas the responses for sensors on Al_2_O_3_ and PET substrates, respectively, were 13.0 and 3.5. The strong response on the paper substrate was due to the rough and porous nature of paper, which led to high porosity and better exposure of the CQD surfaces to the target gas molecules. They also explored the fatigue and bending characteristics of the paper-based gas sensor. Even after 180° bending, the resistance showed almost no changes. Furthermore, the sensor prepared with a Pb to S ratio of 4:1 during synthesis showed a stronger response to NO_2_ gas because of more Pb cations residing on the surface, where the adsorption of NO_2_ molecules was improved, which was beneficial for sensing of NO_2_ gas. In another study, the effect of the PbS QDs film thickness on the NO_2_ gas response was reported [68]. The size of the QDs was ~4 nm and different QD films with thicknesses in the range from 500 nm to 1500 nm were deposited on the sensor substrate. The sensor with a thickness of ~1000 nm showed the best response to NO_2_ gas. NO_2_ has a high oxidation potential, acting as a p-type dopant for PbS, increasing the number of free holes. The highest response was recorded for a sensor with a thickness of ~1000 nm; however, the reasons were not mentioned. 

#### 3.1.5. ZnS QD Gas Sensor

**Figure 8:**
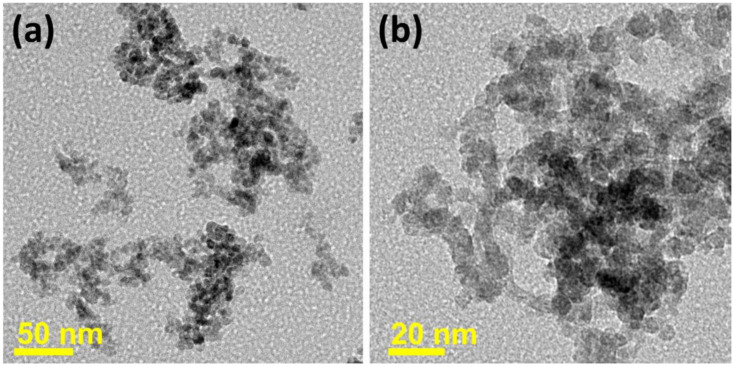
TEM images of (**a**) overlapped ZnS QDs; (**b**) different sizes of ZnS QDs [69].

Mishra et al. [69] synthesized ZnS QDs (Figure 8) for acetone-sensing application. At 174 °C, the sensor indicated selectivity to acetone gas. The strong response to acetone was owing to the high surface area of QDs, which provided large chemisorption of acetone molecules. Furthermore, the rapid response (5.5 s) and recovery time (6.7 s) of ZnS QDs were related to the fast adsorption of oxygen species and their quick interactions with acetone molecules due to the quantum size effects of ZnS QDs.

#### 3.1.6. SnS QD Gas Sensors

SnS has low toxicity and low cost, with a direct and indirect bandgap of 1.0 eV and 1.3 eV, respectively. In this compound, Van der Waal’s force is responsible for bonding Sn and S atoms [70]. The charge exchange between polar gases and SnS is favored because of the anisotropic crystal structure of SnS, making it a good candidate for sensing applications [71,72]. Rana et al. [73] synthesized SnS QDs for ethanol sensing. At 300 °C, it showed a good response and high selectivity to ethanol gas. The ultrafine size, chain-like structure, and appropriate stoichiometry of the SnS QDs improved the response to ethanol gas. 

Wang et al. [74] prepared SnS CQDs for low temperature NO_2_ gas sensing. The sensor exhibited a p-type response and good selectivity to NO_2_ gas. Owing to the paramagnetic nature of NO_2_, upon adsorption, it produces a magnetic dipole beside a surface electric dipole that was generated by the charge. Thus, surface dipoles were formed on the gas sensor, leading to good electron transfer from SnS to NO_2_. Accordingly, a strong response to NO_2_ gas was observed. 

#### 3.1.7. PbCdSe QD Gas Sensor

A new bimetallic Pb_x_Cd_1−x_Se QD (QD) gel consisted of dispersed Pb ionic sites into CdSe crystal revealed a strong response and fast dynamics to NO_2_ gas at 25 °C. The DFT calculation results indicated that Cd sites were responsible for the high NO_2_-sensing output because they offer remarkably higher charge transfer but comparable adsorption energy relative to the Pb sites. The Pb ionic sites acted as the transfer electron density to the neighboring Cd cations, causing them suitable electron donors to NO_2_ gas, improving the gas sensor response [75].

The pristine QD-based gas sensors have merits, such as ease of synthesis and relatively simple operation and mechanism. To realize high-performance gas sensors, it is essential to combine two or three QDs to make heterojunctions and use the synergetic effects between the different materials. The following section provides details of composite QD-based gas sensors. 

## 4. Resistive-Based Gas Sensors on Composite QDs

Not only pristine QDs, but also composite QDs have been used in sensing studies [76,77,78,79,80,81]. Graphene QDs (GQDs) is a 0D nanoscale carbon material, consisting of mono- or a few layers of carbon atoms [82]. GQDs contain carbon dots (CDs) and graphene [83]. GQDs show low toxicity and outstanding conductivity [84]. GQDs also exhibit new features because of the quantum confinement and boundary effects [85]. Thanks to the conjugated π structure, dangling bonds, defects, high surface area, and outstanding electronic mobility, GQDs are promising materials for gas and humidity sensing studies [86,87,88,89,90,91]. Different techniques for synthesis of GQDs have been described in [92,93].

GQD/conducting polymers (CPs) have been used for sensing studies [94,95]. For instance, S and N co-doped GQDs (S, N: GQDs)/polyaniline (PANI) hybrid was used for NH_3_ sensing at 25 °C [96]. The gas sensor had good flexibility and the response increased with the bending angle of substrate. Enhanced response to NH_3_ relative to the pure PANI sensor was related to the intrinsic sensing characteristics of S, N: GQDs. Generally, S, N: GQDs indicated a p-type behavior. Thus, the exposure to NH_3_ gas as an electron donor resulted in a decrease in the number of charge carriers, contributing to the sensing signal. 

**Figure 9:**
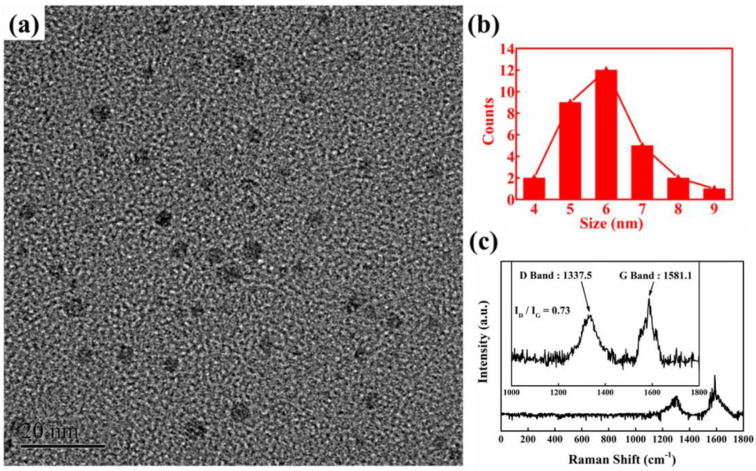
(**a**) TEM image; (**b**) particle size distribution; (**c**) Raman spectrum of N-GQD [97].

Polyaniline (PANI)/N-doped GQD/hollow In_2_O_3_ NF composites are new ternary- sensing compounds synthesized for NH_3_ gas sensing [97]. Figure 9 shows the characterization of N-doped GQD. The response of 20 wt. % N-GQD sensor to 1 ppm NH_3_ was 15.2, which is higher than that of the PANI sensor. 

**Figure 10:**
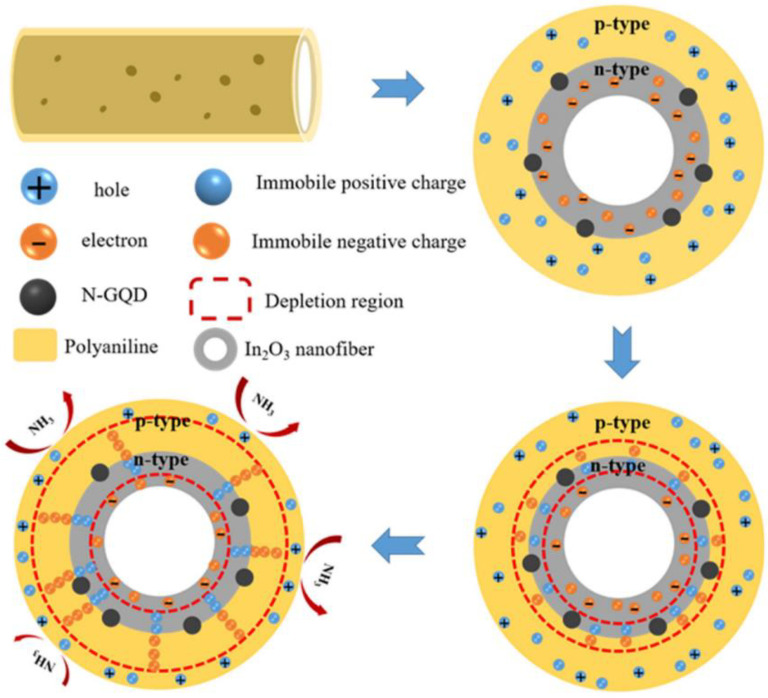
Schematic representation of the sensing mechanism of PANI/GQD/hollow In_2_O_3_ NF composite to NH_3_ gas [97].

Figure 10 presents the sensing mechanism. The sensing material can form chemical bonds on the oxygen-containing defects of the sensing layer. The high surface area gas sensor improved the contact sites with PANI, providing more available sites for NH_3_ molecules. The formation of heterojunctions between the p-type PANI and n-type N-GQD-coated hollow In_2_O_3_ NFs resulted in the formation of an electronic depletion layer along with the production of potential barriers. In an NH_3_ gas atmosphere, the depletion layer thickness was increased, and the sensor resistance was modulated, contributing to the sensing signal. Similar studies based on N-doped GQD have also been reported. PANI/N-doped graphene QDs for NH_3_ gas sensing [98] and PEDOT-PSS,N-doped graphene QDs for NH_3_ sensing [99] are some examples. Also theoretical works have been performed to study interaction of NH_3_ with N-doped GQDs [100]. 

In another study, p–n-GQD-decorated, 3D-ordered macroporous (3DOM) ZnO nanostructures were prepared for acetone sensing. A response of 15.2 to 1 ppm acetone along with fast dynamics was recorded. The creation of a p–n heterojunction between GQDs and ZnO was attributed to the large resistance variations on the sensor. Furthermore, the 3DOM morphology with a hierarchical pore size and the presence of 3D interconnections facilitated high gas diffusion and accessibility and fast carrier flow inside the sensing layer. Moreover, the surface oxygen vacancy amount in the GQD-3DOM ZnO sensor was 40.6%, which was higher than that of the ZnO sensor due to the functionalization of GQDs, resulting in a higher gas adsorption relative to pristine gas sensor [101]. 

A N-GQDs-modified, 3D-ordered macroporous In_2_O_3_ composite was fabricated for NO_2_ sensing applications [102]. The sensor showed an improved response relative to the pristine In_2_O_3_ gas sensor at 100 °C. The generation of N-GQDs/In_2_O_3_ heterojunctions was a major contributor to the sensing signal. The bands of In_2_O_3_ and N-GQDs were bent upon contact and formed an interface depletion layer. In a NO_2_ atmosphere, the depletion layer was expanded at the heterojunction interfaces. Accordingly, a stronger response to NO_2_ for the gas sensor can be achieved. Furthermore, the adding of N-GQDs on In_2_O_3_ provides many active sites for NO_2_ gas molecules allowing more NO_2_ to be adsorbed on the surface of the gas sensor. Moreover, NO_2_ molecules can be preferentially adsorbed on N atoms with a high electron density. 

In another study, MOF-derived ZnO nanopolyhedra/S, N: GQDs/polyaniline (ZnO/S, N: GQDs/PANI) hybrid was synthesized for acetone-sensing studies [103]. The sensor showed a high response to acetone gas. The generation of heterojunction between ZnO and PANI/S, N: GQDs contributed a significant role in enhancing the acetone sensing of the sensor. Furthermore, formation of heterojunctions resulted in a redistribution of charge carriers at the interface of ZnO and PANI/S, N: GQDs, lowering the activation energy for the adsorption of acetone molecules. 

**Figure 11:**
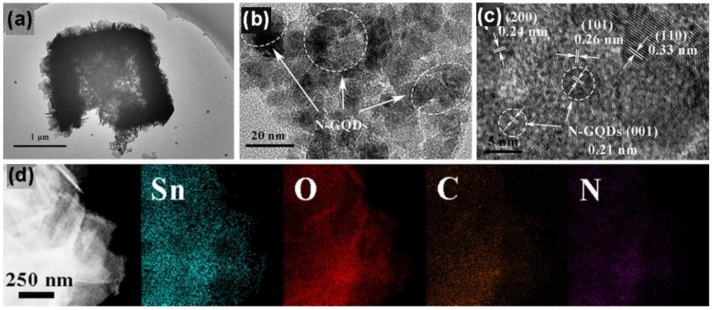
(**a**,**b**) TEM images; (**c**) HRTEM image; (**d**) EDS element mapping of NGQD/Sn_1.5_ [105].

Murali et al. [104] used UV light for activation of an NO gas sensor based on NGQDs-decorated TiO_2_ hybrids. The presence of N-GQDs improved the efficiency of gas and carriers exchange and charge carrier separation, which eventually improved the sensing performance. In another study, [105], N-GQDs were functionalized on the surface of SnO_2_ nanosheets (NG/Sn_x_). The morphology and composition of the NG/Sn_1.5_ sample was studied as shown in Figure 11a–d. GQDs with a lateral size of about 2.7 nm were successfully formed. TEM images of NGQD/Sn_1.5_ demonstrated decoration of N-GQDs on the surface of SnO_2_ nanosheets.

**Figure 12:**
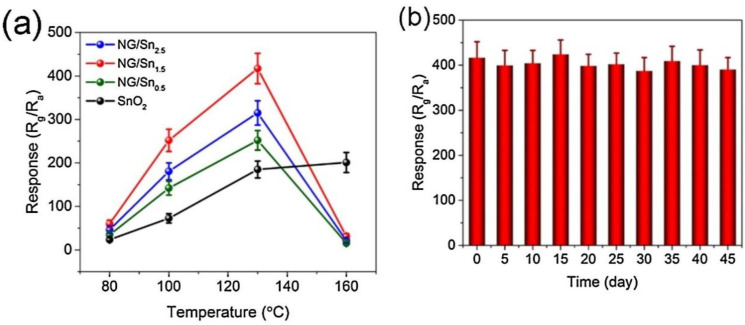
(**a**) Responses of SnO_2_ and NGQD/Snx at various temperatures for 1 ppm NO_2_; (**b**) long-term stability of NGQD/Sn_1.5_ sensor to 1 ppm NO_2_ in 45 days [105].

As shown in Figure 12a, NG/Sn_1.5_ sensor exhibited a response of 417 to 1 ppm NO_2_ gas at 130 °C. Additionally, compared to pristine sensor, the sensing temperature was decreased. Furthermore, Figure 12b demonstrated good stability of gas sensor even after 45 days. 

Due to formation of heterojunctions, the sensor showed a more resistance change compared to pure sensor, and a higher NO_2_ response was recorded. Furthermore, NGQDs have a strong interaction with NO_2_ due to more negative adsorption energy. Also, doped N atoms with high electron density provided plenty of adsorption sites for NO_2_ with strong electrophilic ability. Moreover, the higher electronic partial density of states caused by N doping was beneficial to the electron transfer in the NO_2_ sensing process. Finally, mesopores nature of gas sensor provided many diffusion channels, leading to rapid adsorption of NO_2_. 

Hu et al. [106] fabricated GQDs (2–4 nm)/α-Fe_2_O_3_ composite gas sensor for trimethylamine (TMA) gas sensing. The responses of the sensor to 1000 ppm TMA gas was 1033.0 at 270 °C, which was 187.8 times larger than that of pristine α-Fe_2_O_3_ gas sensor. The sensor also showed a good selectivity to TMA gas. The electron cloud density around N atom in TMA is high; thus, the attractive force between N atom in TMA and Fe^3+^ ion on the surface of the composite facilitated the adsorption of TMA gas. In addition, the bond strengths of C–H, C–C, C–N, C=O, and O–H are 411, 345, 307, 748.2, and 462 KJ/mol, respectively. Thus, the bond energy of C–N in TMA was low, leading to good selectivity of the sensor to TMA. In another work [107], a boron-doped GQD (BGQD)/Ag-LaFeO_3_ p-p sensor was developed for benzene sensing. The sensor exhibited a high response of 17.5 to 1 ppm benzene at 65 °C. The band gap of the BGQDs and Ag-LaFeO_3_ are matched well, enhancing separation of electron-hole pairs and improving the carrier transport ability. The formation of heterojunctions between the BGQDs and Ag-LaFeO_3_ led to the improved carrier-transport ability and reduced the sensing temperature, whereas Ag catalytically improved selectivity to benzene.

GQD-decorated hierarchical SnO_2_ quantum NPs (SnO_2_QNP)/ZnO nanostructures were used for sensing studies. In comparison with pristine ZnO and SnO_2_/ZnO sensors, the GQD-decorated SnO_2_QNP/ZnO nanostructure revealed a high response of 15.9 to 0.1 ppm H_2_S along with fast response/recovery time (14/13 s). The formation of p-n heterojunctions between the p-type GQD/SnO_2_ and ZnO intensified the resistance variation due to the change in oxygen adsorption [108]. Another studies also confirmed the promising effect of GQD for gas-sensing studies [109,110,111,112,113]. 

**Figure 13:**
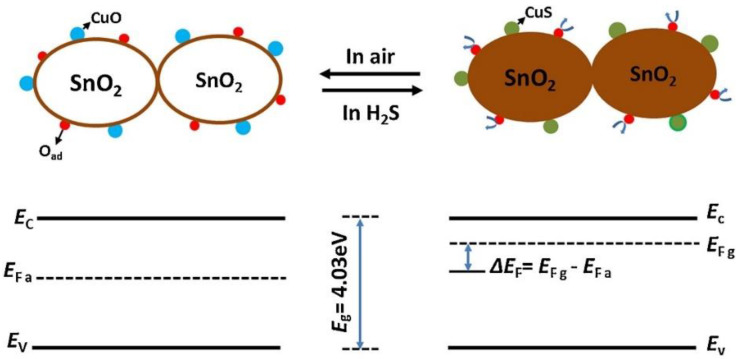
Flat band energy level of the CuO-decorated SnO_2_ QDs in air (**left**) and H_2_S atmosphere (**right**) [114].

Song et al. [114] prepared p-CuO/SnO_2_ QDs for H_2_S sensing studies by treating SnO_2_ QDs with CuCl_2_. At 70 °C, the QD gas sensor exhibited a rapid response of 1755 to 50 ppm H_2_S. Because the SnO_2_ QDs were extremely small, the whole of SnO_2_ QDs were depleted from electrons. Therefore, the extraction of the electrons from the whole crystals and the energy bands of each SnO_2_ QDs were flat without surface barriers for charge transfer at the interfaces of the QDs. Upon exposure to H_2_S gas, electrons are released to the surface of the sensor, leading to a high modulation of the resistance (Figure 13).

In addition, CuO is a well-known material for H_2_S sensing. In H_2_S gas, the semiconducting CuO at the surface of SnO_2_ QDs is transformed to CuS with metallic-like conductivity:

CuO + H_2_S → CuS + H_2_O (3)

Therefore, this transition from a semiconducting state to a conductive state contributes to the sensing signal towards H_2_S gas. 

In another study, porous Co_3_O_4_/SnO_2_ (4.5 nm) QDs heterojunctions were synthesized for xylene sensing studies [115]. The sensitivity of the Co_3_O_4_/SnO_2_QDs to xylene (100 ppm) was three times higher than that of SnO_2_ QDs, demonstrating the beneficial effects of heterojunctions. The Co_3_O_4_/SnO_2_ QDs heterostructures had more Co^2+^ ions for faster Co^2+^/Co^0^ redox reaction in the presence of xylene gas. They had more oxygen vacancies for more active sites and reduced charge transfer resistance on the surface. Furthermore, the size of SnO_2_ QDs was less than the Debye length. Hence, the entire region of SnO_2_ QDs was electron-depleted. Subsequent exposure to xylene gases greatly changes the characteristics of the electron depletion layer that contributed to the final signal. 

**Figure 14:**
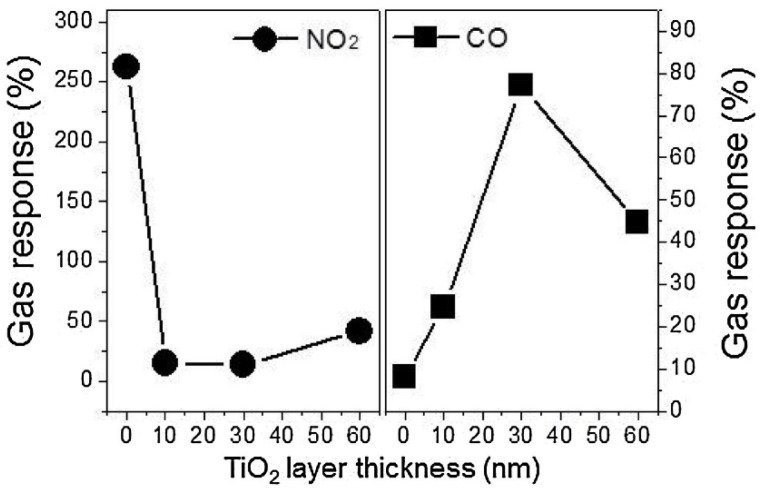
Response of TiO_2_-layer-modified SnO_2_ QDs to NO_2_ and CO gases (1 ppm) at 300 °C [116].

Lee et al. [116] synthesized TiO_2_-layer-modified SnO_2_ QDs. By controlling the number of atomic layer deposition (ALD) cycles, they TiO_2_ layer thickness was set to 10, 30, or 60 nm. At 300 °C, Gas sensing studies revealed that the pristine SnO_2_ QD sensor had the highest response to NO_2_ gas, whereas for CO gas detection, the sensor with a shell thickness of 30 nm exhibited the highest response (Figure 14). 

For modified QDs, heterojunctions were formed and potential barriers were created. The modulation of potential barriers in the presence of target gases had a remarkable role in the appearance of a gas-sensing signal. For CO sensing, the maximum response was realized when the TiO_2_ layer was completely depleted in the ambient air. This occurred for the sensor with a TiO_2_ shell thickness of 30 nm. However, for NO_2_ sensing, due to oxidizing nature of this gas, availability of electrons was an important factor for gas response. For TiO_2_ modified sensors, due to the formation of heterojunctions, there were not enough electrons available to be adsorbed by NO_2_ gas, resulting in a decrease of gas response relative to the pristine SnO_2_ QD gas sensor. 

Transition metal dichalcogenides (TMDs) have a formula of MX_2_ (M = W, Mo, Ti, Zr or Hf and X = S, Se or Te) [117]. They have layered structures that consist of interacting layers of X–M–X bonded together by weak van der Waals forces [118]. Among the TMD family, semiconducting 2D tungsten disulfide (WS_2_) and molybdenum sulfide (MoS_2_) have unique electrical features, such as high mobility of charge carriers and tunable bandgap [119,120]. Compared to 2D MoS_2_, MoS_2_ QDs have a stronger quantum confinement effect and edge effect, which is beneficial for electrical applications [121]. 

CdTe QDs-decorated MoS_2_ nanoworms were fabricated by sputtering for room temperature NO_2_ sensing studies [122]. They indicated a strong response of ∼40 % to 10 ppm NO_2_ at 25°C. Good performance was related to the following: (i) presence of CdTe QDs, which offered more adsorption sites for incoming NO_2_ gas molecules, (ii) the existence of a large surface area, pore interconnectivity and defects, which facilitated the diffusion of NO_2_ molecules, and (iii) the formation of the p-n heterojunctions, with a significant change in the barrier heights in the air and NO_2_ gas. On the other hand, CdTe has cytotoxicity [123]. 

Nanocrystalline ZnO modified with colloidal CdSe QDs were used for room temperature NO_2_ gas-sensing studies [124]. Sensitization by colloidal CdSe QDs was performed using different routes. The CdSe/ZnO sensor obtained using coating of CdSe QDs with a monolayer of mercaptopropionic acid (MPA) with subsequent adsorption on ZnO surface showed the highest sensing capacity for the following reasons: (i) they provide large amounts of CdSe QDs bonded to the ZnO surface, resulting in the transfer of many electrons into ZnO, (ii) the MPA ligand led to closer contact between the ZnO surface and CdSe QDs that improved the charge transfer from CdSe to ZnO, and (iii) p–n heterojunctions were created between CdSe and ZnO, resulting in more modulation of resistance in the porosity of the target gas.

**Figure 15:**
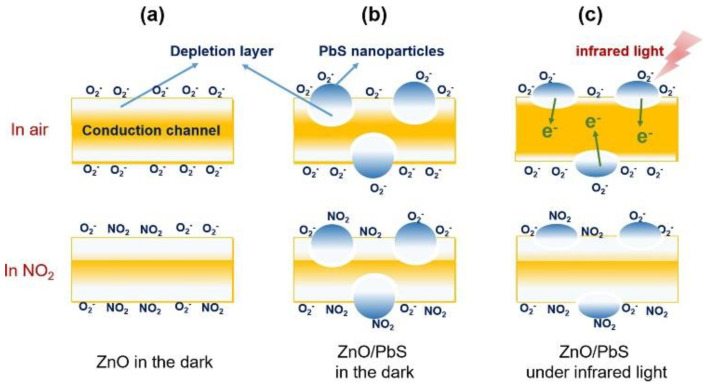
Schematic sensing mechanism of (**a**) ZnO in the dark; (**b**) ZnO/PbS in in the dark; (**c**) under NIR illumination [125].

Chen et al. [125] reported near-infrared (NIR) light illumination (1 mW cm^−2^; λ = 850 nm) for PbS QD (2–5 nm)-decorated (0.5, 2.0 and 5.0 wt%) ZnO nanorods (NRs) for NO_2_ gas sensing. The surface areas of the ZnO NRs (27.1 m^2^ g^−1^) was increased after PbS (2 nm) decoration to 35.3 m^2^ g^−1^. It was related to the presence of PbS QDs with high surface areas. Under 1 mW cm^−2^ NIR illumination, the PdS-decorated (2 nm) ZnO NR sensor showed a response of 123%, which was higher than that of the pristine sensor. For pristine ZnO NRs (Figure 15a, either in the dark or under NIR illumination), modulation of the conduction channel lead to the resistance variations, contributing to the sensing signal. As shown in Figure 15b, when the PbS QDs-decorated ZnO NRs were in a dark chamber, electrons moved from n-type ZnO to p-type PbS, resulting in the formation of electron depletion regions at the interfaces. Thus, when NO_2_ gas is supplied, there is not enough electrons to be adsorbed by NO_2_ gas, resulting in lower responses relative to the pristine gas sensor. However, under NIR activation, PbS QDs were excited and the electrons were injected into the conduction band of ZnO. This resulted in the shrinkage of the electron depletion layer of PbS-decorated ZnO NRs in air (Figure 15c). Therefore, upon injection of NO_2_ gas, there are enough electrons to be adsorbed by NO_2_ gas, resulting in a high response by this gas sensor. 

**Figure 16:**
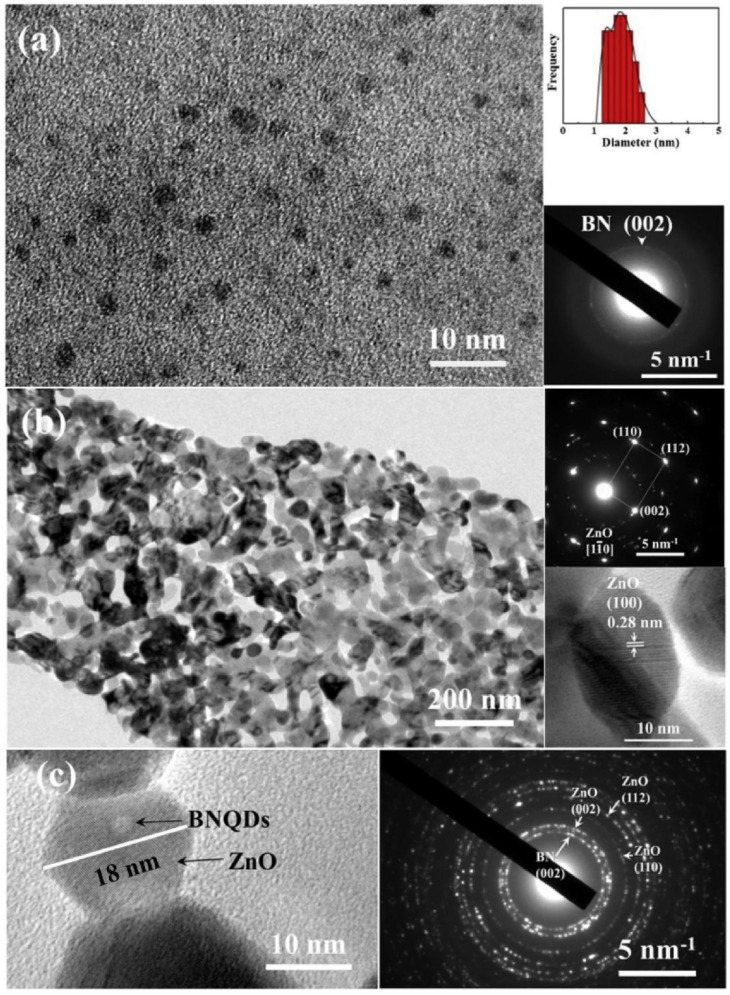
TEM image of (**a**) BN QDs; (**b**) ZnO nanoplates (Insets: Particle size distribution and SAED patterns); (**c**) HRTEM and SAED images of ZnO-BN QDs [126].

Boron nitride QDs (BN QDs)-decorated ZnO nanoplates were synthesized (Figure 16) for benzene, toluene, ethylbenzene, and xylene sensing [126]. The sensing response of ZnO-BN QDs was improved relative to the pristine ZnO gas sensor. A larger surface area can result in enhanced sensing properties, but despite the higher response, the surface area of ZnO-BN QDs (17.84 m^2^/g) was smaller than that of ZnO (24.51 m^2^/g). The BN QDs decoration by hydrothermal method destroyed the porous lamellar structure and reduced the overall porosity of the composite, resulting in a decrease in surface area. Therefore, a higher sensing response was related to the higher adsorption of oxygen molecules on the sensor surface. Because of its lower electronegativity, Zn was attracted by B and N, which in turn reduced the electron scattering effect of Zn, which made more electrons available for enhanced sensing reactions. BN QDs enhanced the adsorption of oxygen species on the sensor surface, leading to more reactions and a higher sensing signal [126].

At the ppb-level detection, TMD-based sensors suffer from poor sensitivity. SnS_2_ QDs/rGO heterostructures were fabricated for ppb level detection of NO_2_ gas sensing at 25 °C. The sensor indicated a strong response of 860% to 125 ppb NO_2_ gas with rapid response (114 s) and recovery times (166 s). The pristine sp^2^ carbon structure of graphene can form outstanding pathways for charge transfer, decreasing the resistance of the gas sensor. In addition, rGO has many residual functional groups and vacancy sites, providing more adsorption sites for incoming gas molecules. Furthermore, A p-n heterojunction can form between SnS_2_ QDs and rGO, and the modulation of heterojunctions contributes to the sensing signal [127].

p-type metal phthalocyanine (MPc) has low conductivity and slow response times for NO_2_ sensing studies. To increase the sensing characteristics of MPc in form of NFs, the GQDs were attached to the surface of MPc NFs through π–π stacking [128]. The high conductivity of GQDs increased the response time of the resulting gas sensor. To decrease the recovery time, a purple laser was applied. Electron (e^−^) and hole (h^+^) pairs were excited by laser on MPc fibers, and the adsorbed NO_2_ molecules underwent transitions from NO_2_^−^ to NO_2_ by taking one hole. This process is very fast; hence, the adsorbed NO_2_ gas molecules can leave the surface of the gas sensor very quickly.

Methylphosphonate (DMMP) is a simulant of sarin nerve gas, thus, its detection is important. GQDs were attached to phthalocyanine (CoPc) derivatives (hexafluoroisopropanol (HFIP) and hexafluorbisphenol A (6FBPA) substituents) for DMMP-sensing studies. At 25 °C, they indicated a good response to DMMP gas, because of the strong hydrogen bonding between the two functional group molecules (HFIP and 6FBPA) of sensors and DMMP gas. Furthermore, GQDs provided good electron channels, where electrons migrated quickly from the host materials to GQDs, producing electrical signals. The response time of the sensor was short because GQDs increased the electrical conductivity by π–π bonding with CoPc derivatives. On the other hand, the sensor showed a slow recovery time. Therefore, laser irradiation was used to accelerate the recovery time. The response of the CoPc-6FBPA-GQD sensor was better than another sensor because of the difference in hydrogen bond energy (HBE) in hydrogen bond complex systems. The HBE of the former sensor (7.8 kcal mol^−1^) was higher than that of the latter sensor (7.7 kcal mol^−1^) [129].

**Figure 17:**
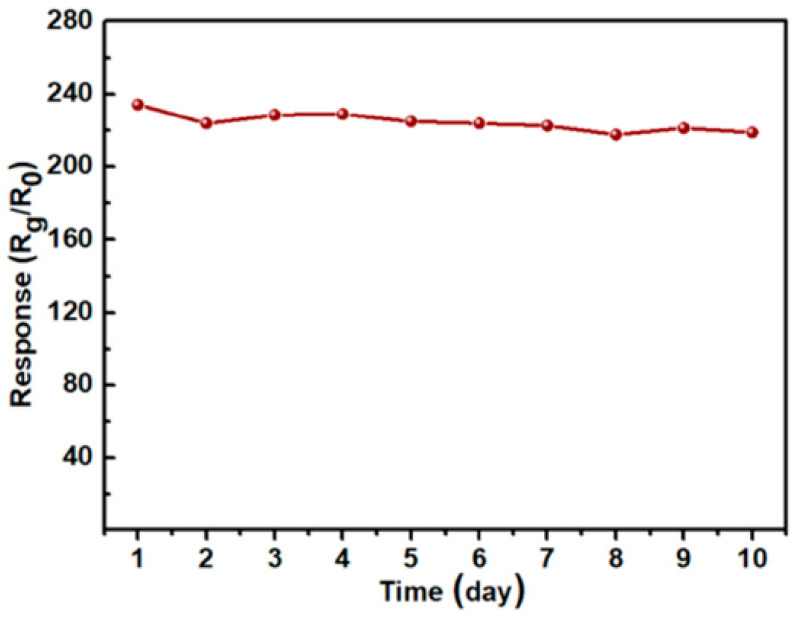
Long-term stability of carbon QD ZnO/gas sensor (100 ppm NO gas/100 °C) [132].

Carbon QDs are mostly used as sensing materials due to their luminescence properties [130]. For example, Wang et al. [131] used functionalized carbon QDs on silica gels for NO_2_ detection where the fluorescence of sensing material was selectively and sensitively quenched by NO_2_ gas. However, there are few researches related to use of carbon QDs as resistive-based gas sensors. For example, carbon QD/ZnO composite was used as an NO gas sensor [132]. At 100 °C, it revealed a high response of 238 to 10 ppm NO gas, which was more than 100 times of that for the pristine ZnO microsphere gas sensor. Additionally, the sensor showed good stability over 20 days as presented in Figure 17. Improved response was related to the porous morphology of the gas sensor with a large surface area and presence of many carbonized hydroxyl groups on the surface of carbon QDs.

The methanol sensing properties of the N-doped carbon QD/Ag-LaFeO_3_ p-n heterojunction were investigated [133]. At 92 °C, the sensor response to 5 ppm methanol was 73. The sensor indicated good selectivity to methanol, which was related to the presence of –COOH groups on the surface, resulting in improved selectivity to methanol. Owing to the high conductivity of carbon QDs, the diffusion of electrons was accelerated, and the overall sensor dynamic was improved. Furthermore, the sensor provided a high surface area, which is highly effective for the adsorption of oxygen and methanol molecules on the surface of the gas sensor. In addition, heterojunctions were formed between the carbon QDs and Ag-LaFeO_3_, resulting in great modulation of resistance.

**Figure 18:**
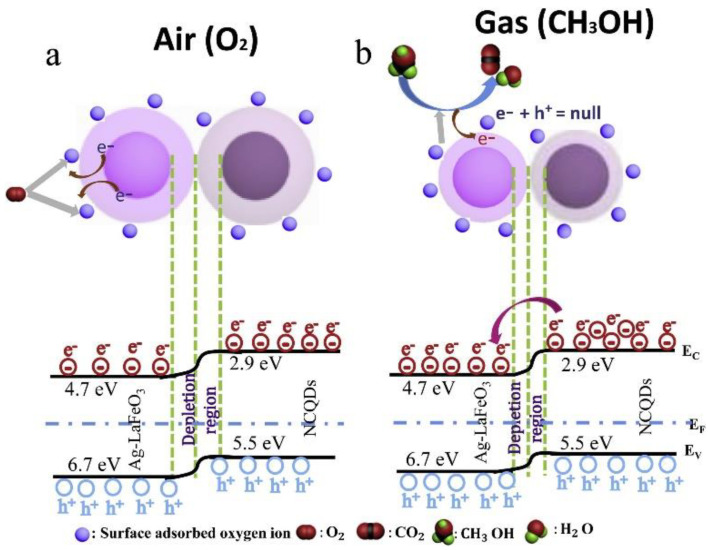
Mechanism of methanol detection by N-doped carbon QD/Ag-LaFeO_3_ gas sensor in (**a**) air and (**b**) CH_3_OH gas [133].

As shown in Figure 18, upon intimate contact between Ag-LaFeO_3_ and carbon QDs, electrons were moved from carbon QDs to Ag-LaFeO_3_ until the Fermi levels were equal on both sides. This resulted in band bending and formation of energy barriers for flow of electrons in the sensor. Upon exposure to methanol gas, the released electrons flowed back to the surface of gas sensor, decreasing the amount of band bending and energy barriers for flow of electrons, resulting in the modulation of the resistance.

In [130], different techniques used for synthesis of carbon QDs have been reviewed.

## 5. Resistive-Based Gas Sensors on Noble Metal Decorated QDs

Noble metals, such as Rh [134], Ru [135], Pt [136], Pd [137], Au [138], and Ag [139], can be decorated on the surface of resistive-based gas sensors to enhance the sensing performance. Unfortunately, only a few studies used noble metals on the surface of metal oxides or metal sulfides for sensing purposes. For instance, Liu et al. [140] prepared Ag-decorated (molar ratio of Ag/Ti = 0%, 1%, 3% and 5%,) TiO_2_ QD gas sensors for room temperature ammonia sensing. Ag-decorated TiO_2_ QDs sensors revealed higher sensitivity and faster dynamics than the pristine sensor. The sensor with 3% Ag showed a strong response of 25.1 to 20 ppm NH_3_. The decorated Ag enhanced the response of the gas sensor to ammonia electronically and catalytically. In another study [141], the Au-decorated ZnO QDs gas sensor showed a higher sensing performance at a lower temperature (35 °C) than pristine ZnO QDs. On the other hand, the selectivity of the gas sensor was poor as it showed a similar response to ethanol and methanol.

Another study reported a room temperature selective CO_2_ gas sensor using ruthenium-decorated tungsten disulfide (Ru-WS_2_) QDs [142]. Ru is a rare earth material with good catalytic activity that is less expensive than Pd and Pt [143]. For the Ru-WS_2_ QD sensor, excellent sensing behavior was observed relative to pristine WS_2_ because of the increase in surface area and breaking of CO_2_ into CO and oxygen species on the surface of Ru. This decreased the number of holes in the Ru-WS_2_ QD sensor, which led to increased resistance.

## 6. Conclusions and Outlooks

This paper discussed the gas-sensing features of different QD-based resistive gas sensors. The most widely used materials in the form of QDs for gas-sensing applications are metal oxides such as SnO_2_ and ZnO, metal sulfides such as PbS, and TMDs such as WS_2_ and GQDs. Due to their extremely fine size, generally, QD-based gas sensors work at low or room temperatures. In particular, the room temperature QD-based gas sensors generally show high sensitivity, high selectivity, and fast dynamics owing to the extremely small size of QDs with a high-surface area and quantum size effects.

There is some considerations related to development of QD-based gas sensors. First, due to their very small sizes, they tend to be agglomerated, which can lead to the instability of gas sensors or decreases in sensing performance. Therefore, development of synthesis methods or post-synthesis methods to have discrete QDs for sensing studies is necessary. Additionally, the current synthesis methods are not able to synthesis the large scale of QDs. Furthermore, exact control of the shape of QDs is difficult. Thus, we need to develop more novel and flexible routes to not only control the size and shape of QDs, but to also produce QDs on large scales.

Based on the literature about the gas-sensing properties of QDs, generally they work at low temperatures. However, when the sensing temperature is still relatively high, there is a danger that QDs will begin to sinter, which will lead to a drift in sensor characteristics. In addition, metal sulfides can be oxidized, especially if an oxidant gas such as NO_2_ is detected. Therefore, it seems that direction in the field of QDs should be towards development of low temperature gas sensors to avoid above-mentioned problems. In most some cases, the researchers have not explored the long term. In addition, in most cases, the stability of a QD-based gas sensor is not presented for long periods and this aspect also requires more study.

Since noble metals can enhance the sensing properties of resistive gas sensors, this aspect needs more study and research. In fact, the effect of noble metal decoration on QD-based gas sensors is less studied. Thus, future studies can be directed to examine the role of noble metals on the surface of QDs. Furthermore, optimization of the amount of noble metals should be studied. The humidity effects on the sensing output as room temperature for QD-based gas sensors is not well-studied. Hence, more information and investigations is needed about different aspects of humidity on the sensing performance of QD-based gas sensors. In some cases, selectivity studies are not performed on QD-based gas sensors [144] and, in this aspect, more research is also necessary. Future directions may include the effect of high-energy beams, such as electron beams or gamma rays on the sensing performance of QD-based gas sensors. The self-heating operation is also another area that needs to be explored in the field of QD gas sensors. Flexible and wearable QD gas sensors also warrant more study and experimentation.

## Figures and Tables

**Table 1:** Exciton Bohr radius of some semiconducting metal oxides used in gas sensor areas.

Material	Exciton Bohr Radius (nm)	Ref.
SnO_2_	~2.7	[35]
ZnO	2.34	[36]
PbS	18	[37]
TiO_2_	1.5	[38]
ZnS	2.5	[39]
SnS	7	[40]
In_2_O_3_	2.38	[41]
